# From the desk of Organizing Secretary

**Published:** 2008-10

I on behalf of organizing committee welcome you all to International Conference of Translational Pharmacology and 41^st^ Annual Conference of Indian Pharmacological Society. The Indian Pharmacological Society is now 41 years old. A sea change has taken place in the last four decades in pharmacology and allied disciplines. However, the vision and mission of the society has largely remained unchanged which is to be relevant, dynamic, and contemporary. Like several drugs become obsolete and antibiotics become resistant and redundant, the technology development and changing needs also necessitate redefining the discipline. The laboratory research in drug development is only worthwhile when it is translated into drugs for masses or drugs for incurable diseases and in this process, the academic institutions, scientific laboratories and drug industry, all play a crucial role.

Let us do the SWOT analysis of our society.

The Indian Pharmacological Society is now the one of the largest scientific professional society in India with over 3000 members. This has representation from diverse disciplines of basic and clinical sciences such as medical pharmacology, veterinary pharmacology, biotechnology regulatory pharmacology, etc. We have members from academic institutions, research laboratories, CROs and regulatory authorities. The members of the society are spread over from rural institutions to the most advanced cities of the developed world. The pharmacologists trained in India, whether working in India or abroad, have contributed significantly to science. India also has the state-of-art laboratories in some government and private sectors. The numbers of publication are also on the rise in high impact factor journals.

We must also be conscious of the weaknesses, which we need to introspect. We have not been able to encash optimally the scientific efforts of Indian scientists put collectively. Certainly, a focused approach and collective efforts where multiple institutions or laboratories work together will have synergistic effect and yield better results. There is a need that few thrust areas are identified and task force constituted which can motivate and invite interested researchers to undertake projects with defined and definitive deliverables. The knowledge and skills must be dynamic with free flow of ideas and skills between scientists and clinicians. It is important that collaborative and exchange programs be promoted so that the scientific professionals are better-trained and equipped with updated knowledge and advanced skills.

The emphasis from classical old pharmacy and animal experimentation has fast changed to molecular pharmacology and then to clinical pharmacology though all the techniques retain their importance. Barrier between clinical and basic research and complex issues involved in conducting clinical research make it difficult to translate new concepts from laboratories to clinic and back again to the bench. Thus, translational pharmacology has become the need of the day.

India has often been referred as growing hub of clinical From the desk of Organizing Secretary research and also a country where experimental in vivo and in vitro data can be outsourced because of competence, expertise and good laboratory practices (GLP) compliance. However, GLP, GMP, and GCP should not only be restricted to regulatory studies, the spirit behind these should also become integral component of day-to-day laboratory and clinical setups.

The Annual conference of Indian Pharmacological Society is one of the effective reflections of its scientific, academic, and social contribution. Not only does it allow us to meet, greet, and eat but it also provides a unique forum for exchange of ideas, offers, and opportunities.

With this background, the International Conference on Translational Pharmacology was also organized along with annual conference of Indian Pharmacological Society with the theme “Concept to Clinic”.

There are several innovative steps taken in this conference. We are conscious of the high risk of failure and attracting criticism in introducing any unconventional thing. Yet we are determined to attempt few changes so as to put renewed energy and direction to the pharmacology science in India.

The information about the conference was largely sent using the Internet. However, the conventional postal method was also used for those who requested. The observation that record number of over 2500 enquiries were received and about 1600 have registered from even remote places in India has alleviated the unfounded apprehension about computer use. Electronic submission of abstract though must have been uncomfortable for many, has forced them to acquire minimum computer proficiency. The often casual approach in submitting abstract has largely been avoided as all the abstracts have been peer reviewed by two external reviewers. Getting the abstracts reviewed by experts was a mammoth task yet it could be achieved because of 24x7 work culture of our team and the great co-operation from abstract reviewers. This first effort brought kudos from several international scientists and editors of other journals. I wish this becomes the regular practice.

The abstracts submitted in the conference could be a good surrogate marker of pharmacology research in India. It can be indicative of the quality, quantity and the areas of research being carried out. It can also serve as a source for identifying strengths and opportunities in different laboratories for exploring collaborative research and importantly to guide financial and strategic support to the institutions which have high potential but proportionally lesser output. The abstract analysis revealed that a record 695 abstracts were received in addition to 120 invited talks in three dozen symposia over 3 days.

The analysis of abstracts indicates a high volume of research in herbal and clinical pharmacology. It was observed that some small centers have submitted excellent research work though few in number. Such talent needs to be identified and supported by the government and the funding agencies.

It is heartening to know the great interest of scientists from all over the globe in Indian pharmacology. In spite of several last minute dropouts of speakers due to unfortunate Mumbai terror incident, we have nearly 100 international scientists, who are attending the conference not only for interaction but with the intent of building a long lasting collaboration with Indian laboratories.

Let us together infuse new enthusiasm in young, not so young, and veteran scientists to make pharmacology a relevant, dynamic, contemporary, and most sought after discipline.

